# Doctors’ Mental Health in the Midst of COVID-19 Pandemic: The Roles of Work Demands and Recovery Experiences

**DOI:** 10.3390/ijerph17197340

**Published:** 2020-10-08

**Authors:** Mohd Fadhli Mohd Fauzi, Hanizah Mohd Yusoff, Rosnawati Muhamad Robat, Nur Adibah Mat Saruan, Khairil Idham Ismail, Ahmad Firdaus Mohd Haris

**Affiliations:** 1 Department of Community Health, Faculty of Medicine, Universiti Kebangsaan Malaysia, Jalan Yaacob Latiff, Bandar Tun Razak, Cheras, Kuala Lumpur 56000, Malaysia; fadhli16288@yahoo.com (M.F.M.F.); adibahsaruan87@gmail.com (N.A.M.S.); khairilidham@gmail.com (K.I.I.); 2 Ministry of Health Malaysia, Federal Government Administrative Centre, Putrajaya 62590, Malaysia; 3 Occupational and Environmental Health Unit, Selangor State Health Department, No 1, Wisma Sunway, Jalan Tengku Ampuan Zabedah C 9/C, Seksyen 9, Shah Alam, Selangor 40100, Malaysia; dr_rosnawati@moh.gov.my; 4 Non-Communicable Diseases Unit, Perak State Health Department, Jalan Koo Chong Kong, Ipoh 30000, Perak, Malaysia; ahmad.firdaus.m3@gmail.com

**Keywords:** depression, anxiety, stress, fatigue, recovery, COVID-19, pandemic, demand, doctors, mental health

## Abstract

The COVID-19 pandemic potentially increases doctors’ work demands and limits their recovery opportunity; this consequently puts them at a high risk of adverse mental health impacts. This study aims to estimate the level of doctors’ fatigue, recovery, depression, anxiety, and stress, and exploring their association with work demands and recovery experiences. This was a cross-sectional study among all medical doctors working at all government health facilities in Selangor, Malaysia. Data were collected in May 2020 immediately following the COVID-19 contagion peak in Malaysia by using self-reported questionnaires through an online medium. The total participants were 1050 doctors. The majority of participants were non-resident non-specialist medical officers (55.7%) and work in the hospital setting (76.3%). The highest magnitude of work demands was mental demand (M = 7.54, SD = 1.998) while the lowest magnitude of recovery experiences was detachment (M = 9.22, SD = 5.043). Participants reported a higher acute fatigue level (M = 63.33, SD = 19.025) than chronic fatigue (M = 49.37, SD = 24.473) and intershift recovery (M = 49.97, SD = 19.480). The majority of them had no depression (69.0%), no anxiety (70.3%), and no stress (76.5%). Higher work demands and lower recovery experiences were generally associated with adverse mental health. For instance, emotional demands were positively associated with acute fatigue (adj. *b* = 2.73), chronic fatigue (adj. *b* = 3.64), depression (adj. *b* = 0.57), anxiety (adj. *b* = 0.47), and stress (adj. *b* = 0.64), while relaxation experiences were negatively associated with acute fatigue (adj. *b* = −0.53), chronic fatigue (adj. *b* = −0.53), depression (adj. *b* = −0.14), anxiety (adj. *b* = −0.11), and stress (adj. *b* = −0.15). However, higher detachment experience was associated with multiple mental health parameters in the opposite of the expected direction such as higher level of chronic fatigue (adj. *b* = 0.74), depression (adj. *b* = 0.15), anxiety (adj. *b* = 0.11), and stress (adj. *b* = 0.11), and lower level of intershift recovery (adj. *b* = −0.21). In conclusion, work demands generally worsen, while recovery experiences protect mental health during the COVID-19 pandemic with the caveat of the role of detachment experiences.

## 1. Introduction

A pandemic of the coronavirus disease 2019 (COVID-19) was declared on March 11, 2020 [1] which was preceded by a declaration of a public health emergency of international concern on January 30, 2020 [2]. The first case of COVID-19 was reported in Wuhan, China in December 2019 [3] which then spread around the world. The first few cases of COVID-19 in Malaysia were imported cases reported on January 25, 2020, involving three Chinese nationals [4,5]. The first Malaysian citizen to contract COVID-19 was reported on 4 February 2020 [4,5]. Following an annual mass religious assembly in Kuala Lumpur which was held between 27 February to 1 March 2020, the cases of COVID-19 in Malaysia drastically increased from mid-March 2020 and peaked in May 2020 [4,5,6]. During the period, Malaysia was one of the top 20 countries with the highest number of COVID-19 cases worldwide.

The COVID-19 pandemic changed the daily routines of each individual worldwide in tandem with efforts to prevent and control the transmission of COVID-19 [6]. The increasing trend of suspected and confirmed cases of COVID-19 required healthcare workers, particularly doctors, to be involved in the management of the COVID-19 pandemic such as contact tracing, diagnosis, treatment, and care of patients with COVID-19 [4,5,6]. In this situation, doctors may face a higher level of work demands related to the mental (such as working on diagnosis as COVID-19 symptoms mimic other mild common diseases), physical (such as prolonged working in complete personal protective equipment attire under hot and humid conditions), temporal (such as managing multiple urgent cases under time constraints) and emotional (such as dealing with patients’ death and dying) context. Those doctors who were not directly involved in the management of COVID-19 cases could also be similarly affected. For instance, they may face increased work demands when limited resources, particularly human resources, are being channeled into the management of COVID-19. In addition, patients who are unrelated to COVID-19 at major hospitals are likely to be transferred to other “non-COVID-19“ hospitals which consequently cause patients‘ influx and increase work demand.

Apart from the increased magnitude of various work demands, it is possible that frontliners, particularly doctors, enjoy limited recovery experiences to recover from their job demands. Recovery experiences refer to the psychological state that people experience related to the activities they pursue during non-work time such as psychological detachment from work, control over leisure time, relaxation and mastery [7]. High work demands potentially spillover into the non-work home domain, causing difficulty in psychologically detaching from work or in controlling their leisure time, consequently resulting in poor recovery [8,9,10]. Being mentally or emotionally attached to work as a consequence of high mental or emotional demands may also make psychological detachment from work difficult during the intershift period [9]. The implementation of movement control order or lockdown could also limit involvement in outdoor physical activities that potentially affect recovery [6]. As a result, it is plausible that doctors do not recover from their ever-increasing job demands, which could lead to multiple adverse consequences.

As a result of increasing work demands and possible poor experiences of recovery, healthcare workers, including doctors, are at risk of developing psychological distress and other mental health symptoms [11]. Multiple studies related to mental health have been conducted among healthcare workers. For instance, Rossi et al. (2020) conducted a cross-sectional study in March 2020 immediately preceding the COVID-19 contagion peak in Italy through an online questionnaire among all healthcare workers in Italy [12]. A total of 1379 healthcare workers completed the questionnaire. They found that 49.38% experienced post-traumatic stress symptoms, 24.73% had symptoms of depression, 19.80% reported symptoms of anxiety and 21.90% experienced high perceived stress [12]. Another study in China involved nearly 4000 healthcare workers using the General Health Questionnaire to assess their mental health status had revealed 40% of them had psychological distress, especially those from Wuhan [13]. This was due to the frequent risk of exposures together with an insufficient number of personal protective equipment [13].

Poor mental health among healthcare workers, particularly doctors, is harmful not only to themselves, but also to their patients, organizations, and healthcare services. For instance, various studies before the emergence of COVID-19 have shown that fatigued doctors are at high risk of having commuting accidents [14], contracting needlestick injury [15], making diagnostic, medical and clinical errors [16,17], and experiencing poor recovery [10]. Fatigue among doctors is also associated with less enjoyment in work [18] and high turnover intention [19]. Other consequences include adverse health and wellbeing, work-life dissatisfaction, low quality of life, job dissatisfaction, and poor skill performance [20]. On the other hand, depressed doctors have been associated with improper medical treatment and adversely affect the attitudes towards patient care [21]. One of the study findings shows that role insufficiency among doctors had the strongest association with depressive symptoms [21]. This was supported by a study reported on the years of services that are shown to has a significant association with depression [22]. Meanwhile, anxiety among doctors was associated with the inappropriate judgment made by the doctors due to emotional exhaustion and reduce sleep quality [23]. As for stress, doctors who are stressed tend to perform lower than their capability resulting in low work productivity and an increase in the frequency of absenteeism [24]. A recent study indicated that stress potentially influences unplanned absenteeism among healthcare workers, which may consequently disrupt the delivery of healthcare services [25].

In Malaysia, most of the COVID-19 cases were handled by the Ministry of Health through public hospitals, district health offices and health clinics [4,5,6]. The work involves healthcare workers, particularly medical doctors, in different settings at public hospitals, clinics, administrative offices, and community fieldwork [4]. Their duty, particularly in the midst of COVID-19 pandemic, is often associated with high-intensity and time-pressured working patterns which can lead to a high risk of mental health problems [6,26]. There has been growing concern about mental health issues among healthcare workers, particularly doctors, following this COVID-19 pandemic such as stress, depression, anxiety, and fatigue [27,28,29]. However, local empirical data on doctors’ mental health are not yet available; as a consequence, the burden of doctors’ mental health in Malaysia is unknown and the intervention program is currently not guided by any empirical evidence. Moreover, there is a limited amount of study that empirically examines the role of work demands and recovery experiences in association with doctors’ mental health in the midst of the COVID-19 pandemic.

Therefore, this study aims to estimate the level of doctors’ mental health, i.e., fatigue (i.e., acute fatigue, chronic fatigue), intershift recovery, depression, anxiety, and stress. This study also aims to explore the role of work demands (physical demand, mental demand, temporal demand, and emotional demand) and recovery experiences (psychological detachment from work, control over leisure time, relaxation, mastery) in association with the level of doctors’ mental health. Table 1 outlines the research questions, objectives, and hypotheses of this current study.

## 2. Materials and Methods

### 2.1. Study Setting and Design

This was a part of a large cross-sectional study conducted among all healthcare workers working at all government health facilities in the state of Selangor, Malaysia. The included facilities were one state health office, 12 public hospitals, nine district health offices (and all health clinics under the administration of respective district health offices), one health office at the international sea port, and one health office at an international airport. The district dental health offices (and all dental clinics) were excluded. The study was conducted in May 2020 immediately following the COVID-19 contagion peak in Malaysia [4,5,6] through an online invitation to all study populations.

### 2.2. Study Population

For this present study, the target population was all medical doctors working at the study location which included house officers, medical officers, and medical specialists. A house officer is a medical doctor with a temporary practice certificate undergoing housemanship training [30]. A medical officer is a medical doctor with a full practice certificate who has completed housemanship training but is not yet a specialist [31]. A medical specialist is a medical doctor registered with the National Specialist Registrar, and can be operationally categorized into clinical specialist (who work in clinical departments in the hospital setting), public health medicine specialist (who work at state health office and district health office), and family medicine specialist (who work at health clinics under the administration of district health office) [31]. There are about 1600 house officers, 3500 medical officers, and 900 specialists working at the study locations. All target population was included as the study population. No specific exclusion criteria were set. All of them were invited through repeated weekly announcement through the Occupational Health Unit at each facility. In addition, they were also invited through WhatsApp, email and verbal reminders among each other. Sample size was calculated based on the precision of 0.05, level of confidence of 95%, and prevalence of depression (24.73%), anxiety (19.80%) and stress (21.90%) from a cross-sectional study among healthcare workers in the midst of the COVID-19 pandemic in Italy [12], and the results were 419, 246, and 264 samples, respectively. All participants participated on a voluntary basis, received information about the procedure of the investigation and gave their online consent before participation.

### 2.3. Study Instruments and Data Collection

Data were collected through an online survey by using self-administered questionnaires in May 2020 for a period of one month. The questionnaires contained multiple parts. The first part is the sociodemographic profile, which is a self-constructed questionnaire that directly asked participants’ sociodemographic profile such as age, gender, marital status, and number of children. There is no identifiable data collected such as name or identity card number. The second part is the occupational profile which is a self-constructed questionnaire that directly asked participants’ occupational profile such as workplace (i.e., hospital, state health office, district health office, health clinic), job title (i.e., house officer, medical officer, clinical specialist, public health medicine specialist, family medicine specialist), and job scope (either directly involved in the management of COVID-19).

The third part is work demands measurement by using a modified NASA Task Load Index (NASA-TLX) [32]. Mental, physical and temporal demands were measured by using three out of six subscales of NASA-TLX [32]. It has been shown that each item can be used as standalone which can help researchers to pinpoint the source of work demand without compromising its sensitivity [33]. As for the emotional demand, the item was self-constructed using the following question: “How much emotional pressure do you feel such as anger, sadness, disappointment and others due to the tasks or task elements occurred throughout the COVID-19 pandemic?”. All four items related to work demands were measured using 11-point Likert scales extending from 0 (low) to 10 (high).

The fourth part is fatigue and recovery assessment which was measured by the Occupational Fatigue Exhaustion Recovery (OFER-15) scale [34]. It is a validated questionnaire consisting of three subscales, i.e., acute fatigue, chronic fatigue and intershift recovery. The acute fatigue subscale captures the inability or unwillingness to engage in non-work activities outside the workplace as a direct consequence of previous work-related activity at the workplace. The chronic fatigue subscale items are designed to capture mental, physical, and emotional components that are characteristics of persistent fatigue. The intershift recovery subscale measures the extent to which the respondent perceives to have recovered from acute work-related fatigue before the next work shift. Each subscale consists of 5 items with a 7-point Likert scale scoring from zero (strongly disagree) to six (strongly agree). Each subscale sums the five items; thus, each subscale may produce a score of 0 to 30. The total score for each subscale is to be divided by 30, followed by multiplication of 100. A higher score denotes a higher level of respective subscales.

The fifth part is depression, anxiety and stress assessment which were measured by using DASS-21 [35,36,37]. Depression Anxiety Stress Scale (DASS) is a globally used screening tool and a qualitative measure of distress along the axes of depression, anxiety and stress [35]. It contained a 21-item, 4-point Likert scale that uses each point to indicate the severity of the individuals’ symptoms over the previous week; the points were “0” (“did not apply”) until “3” (“applied very much or most of the time”) [35,36,37]. A higher score denotes a higher level of respective subscales [35,36,37].

The sixth part is recovery experiences assessment which was measured by using a modified Recovery Experiences Questionnaire [38] containing 4 subscales, namely psychological detachment from work, relaxation, mastery and autonomy. Each subscale consists of 4 items with a 7-point Likert scale from 0 (totally disagree) to 6 (totally agree). The scales have good internal consistency between 0.79 to 0.85 [38].

### 2.4. Data Analysis

Initial data analysis was conducted by using IBM-SPSS version 25 (IBM, New York, NY, USA). Univariable data were presented descriptively. Continuous data were summarized in terms of minimum-maximum, mean and standard deviation. Categorical data were presented as frequencies and percentages. Multiple linear regression analysis was conducted to determine the association between work demands (i.e., mental, physical, temporal and emotional demand) and recovery experiences (i.e., detachment, control, relaxation, and mastery) as independent variables with mental health parameters (i.e., acute fatigue, chronic fatigue, intershift recovery, depression, anxiety, and stress) as dependent variables while controlling for sociodemographic factors (i.e., age, gender, marital status, and status of having children) and job factors (i.e., workplace, job title, and job scope involvement in COVID-19 management). All control and independent variables were initially included, and elimination was performed by the backward method. Data were presented as adjusted regression coefficient (Adj.*b*), 95% CI and *p*-value.

### 2.5. Ethical Consideration

This study was registered with the National Medical Research Register (NMRR-20-1467-55564) and obtained ethical approval from the Medical Research Ethic Committee (KKM/NIHSEC/ P20-1521(4)). Consent for participation was obtained through online medium prior to data collection. The anonymity of participants was ensured by not collecting any identifiable data such as name and identity card number. Since this study involved measurement of mental health status in term of fatigue, recovery, depression, anxiety and stress, participants were given the option on a voluntary basis to be personally contacted by a dedicated psychological first aid team by providing an email address or mobile phone number for further detail assessment and management if they perceive that they require psychological support.

## 3. Results

### 3.1. Participants’ Profile

The number of participants was 1050 doctors. Table 2 demonstrates participants’ sociodemographic and occupational profiles. The majority of participants were female (71.5%), married (60.5%), and had no child (52.9%). Most of them were medical officers (55.7%), worked in hospital settings (76.3%), and had direct involvement in COVID-19 management (66.7%).

### 3.2. Work Demands and Recovery Experiences Profiles

Table 3 demonstrates the work demands and recovery experiences profiles. The highest magnitude of work demands was mental demand (M = 7.54, SD = 1.998) while the lowest was physical demand (M = 6.29, SD = 2.396). Participants reported the highest level of experiences in control over leisure time (M = 15.14, SD = 4.919). Psychological detachment from work was the lowest level of recovery experiences reported by the participants (M = 9.22, SD = 5.043).

### 3.3. Mental Health Parameters

Table 4 demonstrates the mental health profiles. Participants reported a higher acute fatigue (M = 63.33, SD = 19.025) level as compared to chronic fatigue (M = 49.37, SD = 24.473) and intershift recovery (M = 49.97, SD = 19.480). The majority of them had no depression (69.0%), no anxiety (70.3%), and no stress (76.5%).

Table 5 demonstrates the intercorrelation among mental health parameters, work demands, and recovery experiences. Depression, anxiety, and stress were strongly intercorrelated with each other. All the intercorrelations were significant and in the expected direction, except for several intercorrelations involving psychological detachment from work. For instance, the correlation of detachment–chronic fatigue was inversely related, while there was no significant correlation between detachment–intershift recovery, detachment–anxiety, and detachment–stress.

Table 6 demonstrates the association of work demands and recovery experiences with multiple mental health parameters while controlling for sociodemographic factors (i.e., age, gender, marital status, and status of having children) and job factors (i.e., workplace, job title, and job scope involvement in COVID-19 management). Those with higher emotional demand had higher risk of acute fatigue (adj. *b* = 2.73), chronic fatigue (adj. *b* = 3.64), poor intershift recovery (adj. *b* = -2.45), depression (adj. *b* = 0.57), anxiety (adj. *b* = 0.47), and stress (adj. *b* = 0.64). On the other hand, a higher magnitude of physical demand is significantly associated with a higher level of acute fatigue (adj. *b* = 0.47) and lower level of intershift recovery (adj. *b* = −0.53). Similarly, temporal demand is significantly associated with a higher risk of acute fatigue (adj. *b* = 1.19) and chronic fatigue (adj. *b* = 1.02). With regard to recovery experiences, the experiences of control, relaxation, and mastery were significantly associated with multiple mental health parameters in the expected direction. For instance, higher level of relaxation was significantly associated with lower level of acute fatigue (adj. *b* = −0.53), chronic fatigue (adj.*b* = −0.53), depression (adj. *b* = −0.14), anxiety (adj. *b* = −0.11), and stress (adj. *b* = −0.15), and higher level of intershift recovery (adj. *b* = 0.58). The detachment experience was significantly associated with multiple mental health parameters, albeit in the opposite direction of what was expected. Higher level of detachment was significantly associated with higher level of chronic fatigue (adj. *b* = 0.74), depression (adj. *b* = 0.15), anxiety (adj. *b* = 0.11), and stress (adj. *b* = 0.11), and lower level of intershift recovery (adj. *b* = −0.21).

## 4. Discussion

This study aims to estimate the level of doctors’ fatigue, recovery, depression, anxiety, and stress, and determine the role of work demands and recovery experiences in association with the level of doctors’ mental health. In principle, participants had relatively high acute fatigue levels as compared to chronic fatigue and intershift recovery, and generally normal levels of depression, anxiety and stress. This current study also found that mental demand was the highest magnitude of work demand during the pandemic, followed by temporal demand, emotional demand, and physical demand. As for the recovery experiences, participants reported the highest experiences of control over leisure time, but the lowest level of experiences related to psychological detachment from work. Work demands and recovery experiences play significant roles in association with doctors’ mental health in the midst of the COVID-19 pandemic. However, recovery experiences of psychological detachment from work demonstrate a significant association with mental health parameters in the unexpected opposite hypothesized direction.

Our findings on the prevalence of depression, anxiety and stress among doctors were generally similar to the general population in other countries that implemented lockdown measures [39,40,41,42]. Physiologically, this could be due to the differences in exposure to sufficient sunlight during lockdown, which causes a fall in serotonin levels that is associated with emotional disorders such as anxiety and depression [40,43]. Psychologically, it could be due to sudden disruption in life routines, being frequently connected to the internet, avoiding activities due to peer pressure, or economic struggles [44]. However, the reported finding in our study could be underestimated as doctors may refuse to admit that they experience psychological symptoms listed in the questionnaires [45]. Nevertheless, a significant proportion of doctors in our study reported having mild to severe depression, anxiety and stress. This is expected, as working in the midst of the COVID-19 pandemic requires them to work long hours, uphold social and moral responsibility as doctors, and face a high risk of contracting infection for oneself or causing infection to others [26,46,47,48,49].

A quantitative study on fatigue and its recovery specifically among doctors or other healthcare workers is limited. However, the findings on fatigue in the present study are comparable with burnout findings in other studies among healthcare workers worldwide [49,50]. Qualitatively, fatigue can be attributed to the high workload, prolonged wearing of protective gear, and limited recovery opportunity [51]. A study by Sasangohar et al. (2020) listed four potential factors of fatigue, i.e., work-related hazards, huge scaled response, process inefficiencies, and financial constraint [52]. Given the relatively higher burden of fatigue as compared to depression, anxiety and stress among healthcare workers, particularly doctors in our study, more studies related to fatigue are required to determine the antecedents, process, and outcomes of fatigue in the midst of the COVID-19 pandemic. In addition, these findings warrant an urgent, targeted intervention among high-risk groups of frontliners, particularly medical doctors, to prevent fatigue-related consequences [20]. Although many people may interpret fatigue as signs of dedicated doctors, the stakeholders should view it as a sign of failure in resource management [53,54].

Higher work demands are generally associated with a higher risk of having poor mental health which is in line with the conservation of resources theory [55]. For instance, those with higher emotional demand were found to have a higher risk of acute fatigue, chronic fatigue, poor intershift recovery, depression, anxiety, and stress. Based on the conservation of resources theory, work demands use up personal resources such as energy, in which its depletion is consequently manifested as adverse mental health [55]. This finding is consistent with empirical finding among nurses which found that long working hours, which may be correlated with high work demand, was positively related to stress [56]. Nevertheless, it should be noted that the cross-sectional design of this current study was unable to infer causation; and thus, it is unknown which one comes first, either work demand or mental health. Contrary to the above findings, we found that mental demand had no significant association with mental health parameters despite being the highest magnitude type of work demand among participants. It should be worth noting that, statistically speaking, this does not mean there is evidence of no-association between them; instead, it means there is no statistical evidence to support the association. This could be due to the unexamined confounders such as individual cognitive appraisal on mental demand [57,58,59]. In this context, mental demand could be appraised as a hindrance that involves excessive constraints and adversely influences mental health or appraised as a challenge that contributes to personal growth and reward [57,58,59].

As for the recovery experiences, a higher level of control, relaxation and mastery were significantly associated with a lower risk of multiple mental health parameters which is the expected direction. In line with the conservation of resources theory, those with high resources, including a higher level of recovery experiences, have a better tendency to conserve or recover their demand-driven depleted resources [55]. However, our study found that psychological detachment from work had the opposite direction of association with mental health. This could be due to the paradoxical effect of lockdown. Among the drastic interventions to flatten the epidemic curve of COVID-19 cases was movement control order [4]. During this period, healthcare workers, particularly doctors, were regarded as essential professions that were not subjected much to the principle of lockdown. Therefore, during this crisis, being at work seems to be more “enjoyable” and less of a mental health burden compared to being “contained” at home due to the freedom it entails. Perhaps this is the reason why detachment from work is in a positive direction with mental health outcomes; however, this has not been empirically confirmed. Similar to previous discussions, the cross-sectional design of this current study is unable to infer causation. It is possible to postulate that those with poor mental health tend to detach from their work regardless of place and time. This could be explained by the “desperation principle” in the conservation of resources theory in which those with exhausted personal resources will stop involved in the demanding work in order to restore their depleted resources [55].

The strength of this study is in the multicentre setting involving all government health facilities throughout one of the most affected states in Malaysia. This study also simultaneously assesses multiple parameters of mental health immediately following the contagion peak of COVID-19 cases in Malaysia. However, there are several limitations related to the methodology such as the cross-sectional design that is unable to infer causation, and thus, the result should be cautiously interpreted. The use of self-reported questionnaires may introduce social desirability bias and common method bias; however, the use of anonymous surveys may reduce such biases [60].

Based on the emerging questions and limitations discussed above, there are several recommendations for future studies. First, due to the non-significant finding of mental demand as opposed to physical, temporal and emotional demands, we recommend future studies to examine other possible confounders, mediators or moderators related to the work demands during the pandemic crisis such as individual cognitive appraisal. Second, future study should consider longitudinal design to allow causal inference which is important for designing intervention by determining the cause and its respective effect. Third, we recommend researchers who may have comparable data prior to and during the COVID-19 pandemic to evaluate whether there are significant differences in the burden of mental health and its associated factors among healthcare workers, particularly doctors.

## 5. Conclusions

The doctors experienced considerable adverse mental health parameters evidenced by a relatively higher level of acute fatigue compared to chronic fatigue and intershift recovery, but generally normal levels of depression, anxiety, and stress in the midst of the COVID-19 pandemic. High work demands and poor recovery experiences are generally associated with poor mental health parameters; however, poor psychological detachment from work was associated with a better level of several mental health parameters. Future study is warranted to assess causality and evaluate the significant difference in the burden of mental health, work demands, and recovery experience parameters by comparing available data prior and during the COVID-19 pandemic among the comparable study population.

## Figures and Tables

**Table 1:** Overview of research questions, specific objectives, and hypotheses.

Research Questions	Objectives	Hypotheses
RQ1: What is the level of doctors’ mental health during the COVID-19 pandemic?	To estimate the level of doctors’ mental health.	Not applicable (this specific objective was achieved through descriptive analysis and does not involved hypothesis testing analysis)
RQ2: Do work demands and recovery experiences significantly associated with mental health parameters among doctors?	To explore the role of work demands and recovery experiences in association with the level of doctors’ mental health.	H1: Work demands are significantly and positively associated with acute fatigue, chronic fatigue, depression, anxiety, and stress.
		H2: Work demands are significantly and negatively associated with intershift recovery.
		H3: Recovery experiences are significantly and negatively associated with acute fatigue, chronic fatigue, depression, anxiety, and stress.
		H4: Recovery experiences are significantly and positively associated with intershift recovery

**Table 2:** Participants’ profile.

Variables, *n* = 1050	Min.	Max.	*n* (%)	Mean (SD)
Sociodemographic Profile				
Age, in years	24.0	59.0		33.08 (6.965)
Gender				
Female			751 (71.5)	
Male			299 (28.5)	
Marital Status				
Single			401 (38.2)	
Married			635 (60.5)	
Separated / Divorce			14 (1.3)	
Number of Children	0	8		1.02 (1.381)
None			555 (52.9)	
One			183 (17.4)	
Two			164 (15.6)	
Three and beyond			148 (14.1)	
Occupational Profile				
Workplace				
Hospital			801 (76.3)	
Health Clinic			204 (19.4)	
District Health Office			35 (3.3)	
State Health Office			10 (1.0)	
Job Title				
House Officer			305 (29.0)	
Medical Officer			585 (55.7)	
Specialist			160 (15.3)	
Job Scope				
Direct Involvement in COVID-19 Management			700 (66.7)	
No Direct Involvement in COVID-19 Management			350 (33.3)	
Month of Involvement in COVID-19 Management (*n* = 700)				
December 2019			25 (3.6)	
January 2020			68 (9.7)	
February 2020			225 (32.1)	
March 2020			307 (43.9)	
April 2020			75 (10.7)	

**Table 3:** Work demands and recovery experiences profiles

Variables, *n* = 1050	Min.	Max.	Mean (SD)
Work Demands			
Mental Demand	0	10	7.54 (1.998)
Temporal Demand	0	10	7.18 (2.141)
Emotional Demand	0	10	6.77 (2.478)
Physical Demand	0	10	6.29 (2.396)
Recovery Experiences			
Control Over Leisure Time	0	24.0	15.14 (4.919)
Mastery	0	24.0	14.12 (5.483)
Relaxation	0	24.0	13.65 (6.000)
Psychological Detachment from Work	0	24.0	9.22 (5.043)

**Table 4:** Mental health profiles.

Variables, *n* = 1050	Min.	Max.	*n* (%)	Mean (SD)
Acute Fatigue	0.0	100.0		63.33 (19.025)
Chronic Fatigue	0.0	100.0		49.37 (24.473)
Intershift Recovery	0.0	100.0		49.97 (19.480)
Depression	0.0	21.0		3.99 (4.688)
Normal (0–5)			725 (69.0)	
Mild (6–7)			144 (13.7)	
Moderate (8–10)			76 (7.2)	
Severe (11–14)			63 (6.0)	
Very Severe (15+)			42 (4.0)	
Anxiety	0.0	21.0		3.50 (4.325)
Normal (0–4)			738 (70.3)	
Mild (5–6)			83 (7.9)	
Moderate (7–8)			110 (10.5)	
Severe (9–10)			32 (3.0)	
Very Severe (11+)			87 (8.3)	
Stress	0.0	21.0		4.84 (4.681)
Normal (0–7)			803 (76.5)	
Mild (8–9)			100 (9.5)	
Moderate (10–13)			79 (7.5)	
Severe (14–17)			43 (4.1)	
Very Severe (18+)			25 (2.4)	

**Table 5:** Intercorrelation among mental health parameters, work demands, and recovery experiences.

	1	2	3	4	5	6	7	8	9	10	11	12	13	14
1. Acute Fatigue	1													
2. Intershift Recovery	−0.617 **	1												
3. Chronic Fatigue	0.553 **	−0.709 **	1											
4. Depression	0.374 **	−0.480 **	0.613 **	1										
5. Anxiety	0.338 **	−0.397 **	0.527 **	0.827 **	1									
6. Stress	0.405 **	−0.452 **	0.583 **	0.878 **	0.869 **	1								
7. Detachment	−0.067 *	0.035	0.071 *	0.071 *	0.050	0.025	1							
8. Control	−0.252 **	0.348 **	−0.303 **	−0.293 **	−0.262 **	−0.271**	0.275 **	1						
9. Relaxation	−0.338 **	0.345 **	−0.279 **	−0.289 **	−0.245 **	−0.290 **	0.413 **	0.602 **	1					
10. Mastery	−0.352 **	0.328 **	−0.312 **	−0.308 **	−0.227 **	−0.267 **	0.003	0.395 **	0.356 **	1				
11. Mental Demand	0.341 **	−0.203 **	0.239 **	0.151 **	0.144 **	0.192 **	−0.096 **	−0.013	−0.079 *	−0.049	1			
12. Physical Demand	0.373 **	−0.299 **	0.314 **	0.194 **	0.224 **	0.220 **	−0.042	−0.093 **	−0.118 **	−0.057	0.488 **	1		
13. Temporal Demand	0.451 **	−0.311 **	0.354 **	0.222 **	0.211 **	0.263 **	−0.111 **	−0.080 **	−0.178 **	−0.122 **	0.780 **	0.560 **	1	
14. Emotional Demand	0.556 **	−0.440 **	0.501 **	0.379 **	0.333 **	0.413 **	−0.068 *	−0.183 **	−0.233 **	−0.185 **	0.617 **	0.524 **	0.673 **	1

Significance level: * < 0.05; ** < 0.01.

**Table 6:** Association of work demands and recovery experiences with multiple mental health parameters *.

Variables, *n* = 1050	Acute Fatigue		Chronic Fatigue		Intershift Recovery		Depression		Anxiety		Stress	
	Adj. *b* (95% CI)	*p*-value	Adj. *b* (95% CI)	*p*-value	Adj. *b* (95% CI)	*p*-value	Adj. *b* (95% CI)	*p*-value	Adj. *b* (95% CI)	*p*-value	Adj. *b* (95% CI)	*p*-value
**Work Demands**												
Mental Demand												
Physical Demand	0.47 (0.01, 0.92)	0.044			−0.53 (−1.00, −0.07)	0.024						
Temporal Demand	1.19 (0.60, 1.77)	<0.001	1.02 (0.30, 1.74)	0.006								
Emotional Demand	2.73 (2.24, 3.23)	<0.001	3.64 (3.01, 4.27)	<0.001	−2.45 (−2.91, −1.99)	<0.001	0.57 (0.47, 0.67)	<0.001	0.47 (0.37, 0.57)	<0.001	0.64 (0.54, 0.74)	<0.001
**Recovery Experiences**												
Detachment			0.74 (0.49, 0.99)	<0.001	−0.21 (−0.42, −0.01)	0.040	0.15 (0.10, 0.21)	<0.001	0.11 (0.06, 0.16)	<0.001	0.11 (0.06, 0.17)	<0.001
Control			−0.42 (−0.73, −0.12)	0.006	0.32 (0.07, 0.58)	0.012	−0.08 (−0.15, −0.02)	0.010	−0.09 (−0.15, −0.02)	0.007		
Relaxation	−0.53 (−0.71, −0.34)	<0.001	−0.53 (−0.79, −0.27)	<0.001	0.58 (0.36, 0.79)	<0.001	−0.14 (−0.20, −0.09)	<0.001	−0.11 (−0.16, −0.06)	<0.001	−0.15 (−0.21, −0.10)	<0.001
Mastery	−0.68 (−0.86, −0.51)	<0.001	−0.49 (−0.72, −0.25)	<0.001	0.46 (0.27, 0.65)	<0.001	−0.11 (−0.16, −0.06)	<0.001			−0.08 (−0.13, −0.03)	0.003
**Adjusted R^2^**	0.44		0.43		0.37		0.30		0.23		0.29	

* Multiple linear regression with work demands and recovery experiences as independent variables and mental health parameters as dependent variables while controlling for sociodemographic factors (i.e., age, gender, marital status, and status of having children) and job factors (i.e., workplace, job title, and job scope involvement in COVID-19 management.
